# Phagocytosis of mast cell granules results in decreased macrophage superoxide production

**DOI:** 10.1155/S0962935195000652

**Published:** 1995-11

**Authors:** Bobby A. Shah, Yuai Li, Daniel J. Stechschulte, Kottarappat N. Dileepan

**Affiliations:** 1 Division of Allergy Clinical Immunology and Rheumatology Department of Medicine University of Kansas Medical Center Kansas City KS 66160-7317 USA

## Abstract

The mechanism by which phagocytosed mast cell granules (MCGs) inhibit macrophage superoxide production has not been defined. In this study, rat peritoneal macrophages were co-incubated with either isolated intact MCGs or MCG-sonicate, and their respiratory burst capacity and morphology were studied. Co-incubation of macrophages with either intact MCGs or MCG-sonicate resulted in a dose-dependent inhibition of superoxide- mediated cytochrome c reduction. This inhibitory effect was evident within 5 min of incubation and with MCG-sonicate was completely reversed when macrophages were washed prior to activation with PMA. In the case of intact MCGs, the inhibitory effect was only partially reversed by washing after a prolonged co-incubation time. Electron microscopic analyses revealed that MCGs were rapidly phagocytosed by macrophages and were subsequently disintegrated within the phagolysosomes. Assay of MCGs for superoxide dismutase (SOD) revealed the presence of significant activity of this enzyme. A comparison of normal macrophages and those containing phagocytosed MCGs did not reveal a significant difference in total SOD activity. It is speculated that, although there was no significant increase in total SOD activity in macrophages containing phagocytosed MCGs, the phagocytosed MCGs might cause a transient increase in SOD activity within the phagolysosomes. This transient rise in SOD results in scavenging of the newly generated superoxide. Alternatively, MCG inhibition of NADPH oxidase would explain the reported observations.


Mediators of Inflammation 4, 406–412 (1995)

### Phagocytosis of mast cell granules results in decreased macrophage superoxide production

Bobby A. Shah, Yuai Li, Daniel J. Stechschulte and Kottarappat N. Dileepan[CA]

Division of Allergy, Clinical Immunology and Rheumatology, Department of Medicine, University of Kansas Medical Center, Kansas City, KS 66160-7317, USA

[CA]Corresponding Author

**THE mechanism by which phagocytosed mast cell granules (MCGs) inhibit macrophage superoxide production has not been defined. In this study, rat peritoneal macrophages were co-incubated with either isolated intact MCGs or MCG-sonicate, and their respiratory burst capacity and morphology were studied. Co-incubation of macrophages with either intact MCGs or MCG-sonicate resulted in a dose-dependent inhibition of superoxide-mediated cytochrome c reduction. This inhibitory effect was evident within 5 min of incubation and with MCG-sonicate was completely reversed when macrophages were washed prior to activation with PMA. In the case of intact MCGs, the inhibitory effect was only partially reversed by washing after a prolonged co-incubation time. Electron microscopic analyses revealed that MCGs were rapidly phagocytosed by macrophages and were subsequently disintegrated within the phagolysosomes. Assay of MCGs for superoxide dismutase (SOD) revealed the presence of significant activity of this enzyme. A comparison of normal macrophages and those containing phagocytosed MCGs did not reveal a significant difference in total SOD activity. It is speculated that, although there was no significant increase in total SOD activity in macrophages containing phagocytosed MCGs, the phagocytosed MCGs might cause a transient increase in SOD activity within the phagolysosomes. This transient rise in SOD results in scavenging of the newly generated superoxide. Alternatively, MCG inhibition of NADPH oxidase would explain the reported observations.**

**Key words:** Macrophages, Mast cell granules, Phagocytosis, Superoxide, Superoxide dismutase

### Introduction

Macrophages exhibit a wide array of immunological functions. These include participation in the natural resistance against microorganisms, inhibition of tumour cell proliferation and regulation of the immune system via antigen presentation and cytokine production.[1,2] One of the active mechanisms by which macrophages kill microorganisms is by producing reactive oxygen radicals.[3–6] When phagocytic cells encounter immunoglobulin G coated organisms or identify certain soluble agents of the pathogen, they undergo respiratory burst and generate superoxide and other reactive oxygen metabolites.[7] Although production of superoxide and the metabolites such as $H_2O_2$ and hydroxyl radicals are key steps in the oxidative damage of the invading pathogens, over-production of these radicals can damage the host tissues.[8] In order to protect the host cells from any unwarranted oxidative destruction, the cells themselves are provided with anti-oxidant enzymes such as superoxide dismutase (SOD), catalase and glutathione peroxidase. In the event of unchecked production of reactive oxygen radicals, the scavenging enzymes present in the host cells may be insufficient for protection. Under such circumstances, cell to cell communication and translocation of the detoxifying components between cell types may be vital for the integrity of the tissues. In this study, we investigated whether such a co-operative phenomenon for the regulation of reactive oxygen metabolism exists between mast cells and macrophages.

Previous reports from this laboratory have shown that degranulating mast cells and isolated mast cell granules (MCGs) could rapidly interact with macrophages[9,10] and eosinophils[11] and inhibit superoxide-mediated cytochrome c reduc-

© 1995 Rapid Science Publishers tion. Two explanations for the inhibitory effect of MCGs are possible: (a) decreased generation of superoxide resulting from the inhibition of the respiratory burst enzyme NADPH oxidase; or (b) enhanced scavenging of the generated superoxide by the MCG components. The present study was undertaken as an attempt to unravel the mechanism of inhibition on macrophage superoxide production by mast cell granule components.

### Materials and Methods

*Animals:* Two- to 3-month old male Sprague–Dawley rats (SASCO, Omaha, NE) were used for harvesting serosal mast cells and macrophages. The animals were housed in microbarrier cages with free access to food (Purina rodent chow) and purified water. They were euthanized using Metophane prior to lavage of the thoracic and peritoneal cavities.

*Collection and isolation of macrophage and mast cells:* Macrophages and mast cells were collected from the serosal cavities of rats[10] by lavage of the peritoneal and thoracic cavities of each animal with 15 ml Tyrode's buffer containing 0.1% gelatin (TG) and 50 μg/ml heparin.[11–13] The serosal cells from all animals were pooled and sedimented by centrifugation at 400 × **g** for 15 min at room temperature. The cells were washed twice with TG and resuspended in TG. Two-ml cell suspensions (between 6 to 8 × $10^7$ cells) were layered on 4 ml of 22.5% (w/v) metrizamide (density 1.125 g/ml) and centrifuged at 200 × **g** for 15 min. The macrophages at the interface were collected, washed twice, and then resuspended in TG. Macrophages isolated in this manner had little or no contamination with mast cells. The mast cells in the pellet were collected, washed twice, and resuspended in calcium- and magnesium-free TG containing 2.5 mM EDTA. Mast cells isolated by this procedure exceeded 90% in purity and viability.

*Preparation of MCG and MCG-sonicate:* MCGs were prepared from mast cells by controlled sonication and sucrose gradient centrifugation as described previously.[14,15] Briefly, purified mast cells were suspended in 2 ml $Ca^{2+}$ and $Mg^{2+}$-free TG containing 2.5 mM EDTA (EDTA–TG). The cell suspension was sonicated for 15 s, cooled for 30 s on ice, and resonicated for 15 s at a power setting of 2.5 with a microtip sonicator. The disrupted cells were incubated at 30°C for 15 min. After being vortexed for 1 min at medium speed, the suspension was layered over 2 ml of 0.34 M sucrose and centrifuged at 50 × **g** for 10 min at 4°C. The upper layer containing mast cell granules was collected and centrifuged at 1800 × **g** for 20 min at 4°C. The resulting pellet consisting of a homogeneous preparation of granules was washed twice with EDTA–TG and resuspended in a suitable volume of TG. The recovery of mast cell granules isolated by this procedure ranged from 50% to 70% on the basis of the histamine content of the starting mast cells. MCG-sonicate, the disrupted MCGs, was prepared from MCG by sonicating 2 × 20 s at maximum power.

*Superoxide production:* Superoxide production was assayed by means of the superoxide dismutase-sensitive reduction of cytochrome c[5] with minor modifications.[10,16] The reaction mixture contained 2–3 million macrophages, 50 nmol cytochrome c, and when present, the indicated concentrations of MCGs or MCG-sonicate, in a final volume of 1 ml TG or Hank's balanced salt solution (HBBS). Control assays contained 250 units of superoxide dismutase in addition to the standard ingredients. The ingredients were preincubated for 5 min at 37°C and macrophage superoxide production was initiated by addition of phorbol myristate acetate (PMA, 200 ng/ml). The superoxide-mediated cytochrome c reduction was measured by monitoring the absorbance at 550 nm on a Beckman DU 70 spectrophotometer.

*Superoxide dismutase activity:* Superoxide dismutase activity in MCG extract was assayed as described previously.[11,17] The assay system contained 50 mM of potassium phosphate (pH 7.8), 0.1 mM xanthine, 0.02 mM cytochrome c, 0.1 mM EDTA, xanthine oxidase sufficient to generate an absorbance change of 0.035 to 0.04 per min, and selected doses of MCG extract or commercial SOD in a final volume of 1.0 ml. After 5 min preincubation at 30°C the reaction was started by addition of xanthine oxidase and the superoxide-mediated cytochrome c reduction was continuously monitored at 550 nm for 5 min on a Beckman DU 70 spectrophotometer.

*Protein assay:* Protein contents were quantified by employing BCA protein assay kit from Pierce Chemical Co, (Rockford, Il).

*Statistics:* One-way ANOVA and the Student–Newman–Kuel test were used for the analyses of the data. All values given are expressed as the mean ± S.E.M. $p < 0.05$ was considered significant.

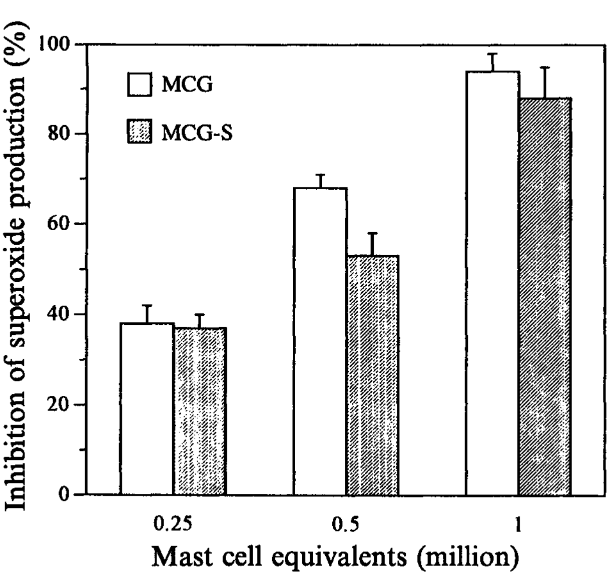

FIG. 1. Effect of MCG and MCG-sonicate on macrophage superoxide-mediated cytochrome c reduction. 2.5 × $10^6$ macrophages were incubated with the indicated amount of MCG or MCG-sonicate for 5 min at 37°C. Superoxide production by macrophages was initiated by the addition of 0.2 µg/ml PMA, and cytochrome c reduction was assayed as described in the Material and Methods section. Data presented are % inhibition of control macrophages activated with PMA. Values are mean ± S.E.M., $n=5$. MCG-S: MCG-sonicate.

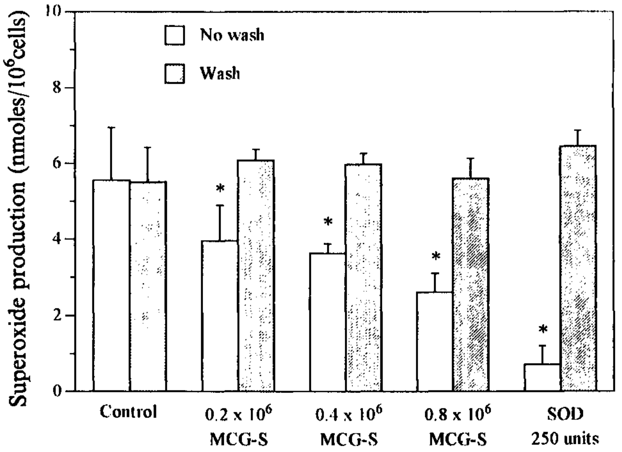

FIG. 2. Effect of MCG-sonicate or SOD on macrophage superoxide production. 3 × $10^6$ macrophages were incubated with the indicated amount of MCG-sonicate or SOD for 30 min at 37°C. Macrophages were then washed and resuspended in HBSS for superoxide assay after activation with PMA (0.2 µg/ml). The dose of MCG-sonicate is expressed in mast cell equivalents. Values are mean ± S.E.M., $n=6$. $^*p < 0.05$ *vs.* control. MCG-S: MCG-sonicate.

### Results

*Effect of MCG and MCG-sonicate on macrophage superoxide-mediated cytochrome c reduction:* Co-incubation of macrophages with intact MCGs or MCG-sonicate for 5 min prior to activation with PMA resulted in dose-dependent inhibition of superoxide-mediated reduction of cytochrome c (Fig. 1). The inhibitory effect of MCGs on superoxide-mediated cytochrome c reduction ranged from 40 to 100% for MCG doses of 0.25 to 1.00 × $10^6$ mast cell equivalents. The inhibitory effect of MCGs was comparable whether intact MCGs or MCG-sonicate was used.

*Effect of soluble MCG products or SOD on macrophage generated superoxide production:* The inhibitory effects of MCG factors on macrophage superoxide generation could be due to inhibition of the respiratory burst enzyme NADPH oxidase or due to scavenging of the generated superoxide. In order to identify the effect of MCGs, macrophages were incubated with buffer or varying doses of MCG-sonicate or SOD for 30 min. Each macrophage preparation was then divided into a washed (to remove MCG-sonicate or SOD) and unwashed preparations, and resuspended in HBSS. Superoxide production was then assayed according to the standard protocol after activation with PMA. As shown in Fig. 2, macrophages incubated with MCG-sonicate or SOD and then activated with PMA produced significantly lower levels of superoxide. The inhibition by MCG-sonicate was dose-dependent and ranged from 34% to 67% with varying doses of MCG-sonicate. On the other hand, macrophages that were washed after pre-treatment with MCG-sonicate or SOD produced the same amounts of superoxide as the control macrophages. Washing of the cells alone did not alter the capacity of macrophages for superoxide production.

*Association of the effect of MCGs with phagocytosis:* The finding that soluble MCG extract was capable of inhibiting superoxide-mediated cytochrome c reduction only if present at the time of macrophage activation prompted us to evaluate whether the inhibition was associated with internalization of the granule. Macrophages were incubated with MCGs for 5 or 30 min. Each of the cell suspensions was washed twice and sedimented by slow centrifugation. The pellets of macrophages thus separated from free MCGs were resuspended in HBSS for superoxide production assays after activation with PMA. Macrophages were also activated with PMA without removing free MCGs. As shown in Fig. 3, incubation of macrophages with intact MCGs for 5 min (Fig. 3, upper panel) resulted in complete inhibition of superoxide-mediated cytochrome c reduction and washing resulted in a 21% recovery. In the case of macrophages incubated with MCGs for 30 min without removal (Fig. 3, lower

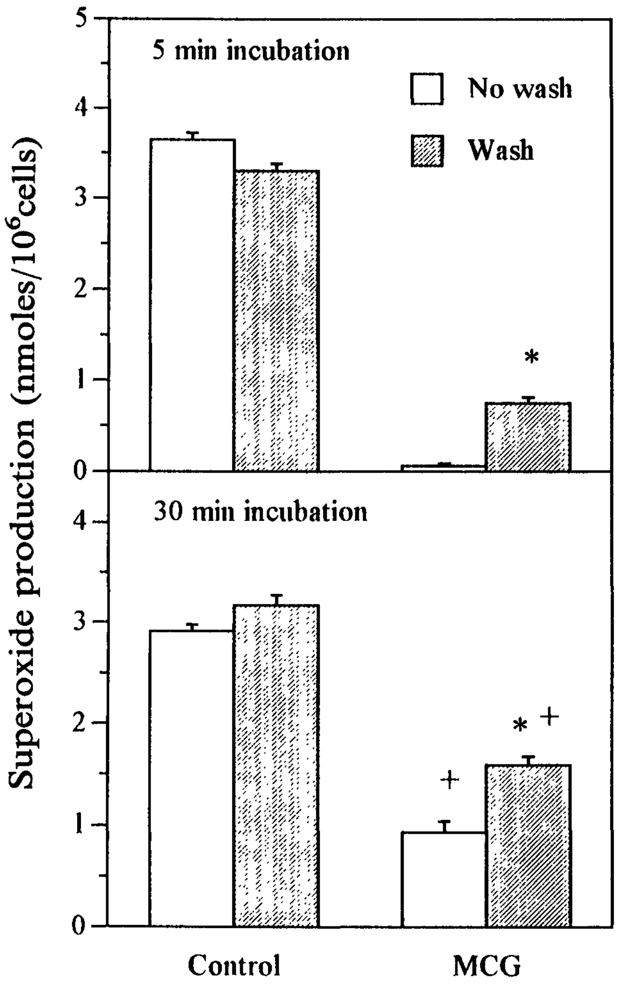

FIG. 3. Effect of wash and incubation time on macrophage superoxide production inhibited by MCG. $2.5 \times 10^6$ macrophages were incubated with $0.8 \times 10^6$ MCG of mast cell equivalents for 5 min (upper panel) or 30 min (lower panel) at 37°C. The cells were washed twice and resuspended in HBSS for superoxide assay as described in the Materials and Methods section. Values are mean $\pm$ S.E.M., $n = 5$. $^*p < 0.05$ when compared to wash cells; $p < 0.05$ when compared to cells incubated for 5 min.

panel), the inhibitory effect was of lesser magnitude (78% *vs.* 98%). Washing of the cells resulted in partial recovery of superoxide release to about 50% of the control release and was significantly greater than that observed after 5 min of stimulation. The sucrose density gradient centrifugation and washing procedure did not affect the capacity of macrophages to generate superoxide.

*Electron microscopic analyses of macrophages for MCG phagocytosis and degradation:* The finding that washing of macrophages prior to their activation with PMA could completely reverse the inhibitory effect of MCG-sonicate and not that of intact MCGs suggests that MCGs have been internalized and were not removed by washing. Furthermore, the results of Fig. 3 suggest that the reversal of the MCG effect by washing was of higher magnitude if macrophages and MCGs were incubated for longer time (30 min) than those incubated for a shorter period (5 min). Therefore, to assess the association between MCG uptake and the inhibition of superoxide release, and to determine the fate of phagocytosed MCGs, macrophages were examined by using electron microscopy. In this study, macrophages preincubated with MCGs for 10 min and purified by sucrose density gradient centrifugation,[10] were incubated for 0, 15 and 30 min and examined by transmission electron microscopy. Figure 4 shows that MCGs were rapidly taken up by macrophages into the phagosomes within 10 min (Fig. 4B). The phagocytosed MCGs were gradually disintegrated within 30 min (Fig. 4C and 4D).

*Superoxide dismutase activity in MCGs:* Since the pre-treatment of macrophages with MCG-sonicate for up to 30 min could be reversed by washing, this effect is presumably due to the superoxide dismutase present in the MCGs. In order to confirm this, superoxide dismutase activity was assayed in MCG-sonicate. As evident from Table 1, addition of MCG-sonicate inhibited superoxide-mediated cytochrome c reduction in a dose-dependent fashion when assayed using xanthine and xanthine oxidase. Addition of commercially available SOD also showed a dose-dependent inhibition of xanthine oxidase-mediated cytochrome c reduction.

Addition of other MCG components such as histamine and serotonin at concentrations up to 1 mM and 0.1 mM respectively, or heparin (10 μg/ml) did not affect macrophage superoxide-mediated cytochrome c reduction (data not shown).

*Superoxide dismutase activity in control and macrophages with phagocytosed MCGs:* In order to examine if macrophage phagocytosis of MCGs resulted in an increase in intracellular SOD activity, total SOD activity was assayed in Triton X-100 extracts of control and MCGs-treated macrophages. As evident from Fig. 5, there was no significant difference in total intracellular SOD activity between control macrophages and those containing phagocytosed MCGs even though there is an apparent increase in the macrophage/MCG preparations.

### Discussion

Increased numbers of mast cells have been shown to be present in many inflammatory dis-

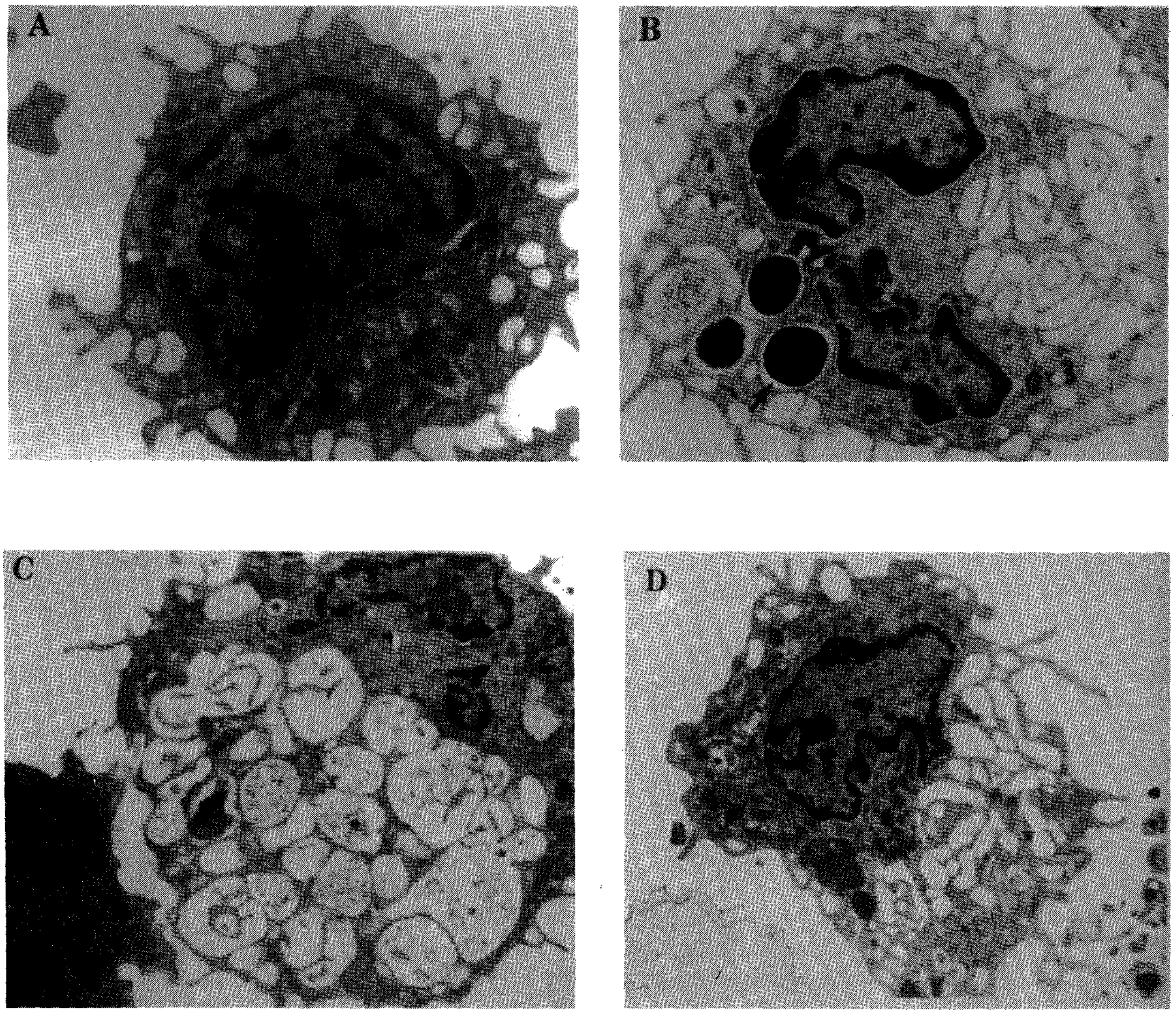

FIG. 4. Electron photomicrographs of macrophages with phagocytosed MCG. Macrophages were preincubated with MCGs for 10 min at 37°C and were purified by sucrose density gradient centrifugation followed by washing with Tyrodes buffer. Aliquots of the macrophage suspensions were incubated for 0, 15 or 30 min. The cells were fixed in 2% gluteraldehyde and examined by transmission electron microscopy. Normal macrophages (A); macrophages with phagocytosed MCG incubated for 0 min (B); 15 min (C) and 30 min (D). Original magnification ×4800. Arrow indicates MCG.

orders.[18–21] However, it is not clear whether mast cells play a pro-inflammatory or an anti-inflammatory role. The interaction of mast cell products with mononuclear cells to produce a factor enhancing prostaglandin $E_2$ synthesis has been reported.[22] Although the fate of the exocytosed MCGs from mast cells is not well understood, phagocytosis of MCGs by macrophages[23] and fibroblasts[24] has been documented. Interaction of MCGs with macrophages has been shown to alter a variety of macrophage function including superoxide production,[10] Fcγ receptor-mediated phagocytosis,[25] tumour cell killing and nitric oxide production.[15] However, a direct relationship between phagocytosis of MCGs and altered macrophage functions has not been shown.

The present study indicates that isolated MCGs are capable of interacting with macrophages and inhibiting superoxide release as determined by cytochrome c reduction. The inhibitory effect was comparable whether intact MCGs or MCG-sonicate was used. The effect was concentration dependent and complete inhibition of superoxide-mediated reduction of cytochrome c could be seen when $1 \times 10^6$ mast cell equivalent of MCGs were present in incubations containing up to $5 \times 10^6$ macrophages. The effect, at least in part, was due to the dismutation of superoxide generated by the respiratory burst enzyme NADPH oxidase. This conclusion is based on the presence of SOD in the MCGs (Table 1) and the finding that the effect of MCG-sonicate on mac-

**Table 1.** Superoxide dismutase activity in mast cell granules

| Addition to xanthine-xanthine oxidase | nmoles of superoxide generated per min | % inhibition |
|---|---|---|
| None | 7.72 | - |
| MCG-sonicate $10^3$ | 7.26 | 6.0 |
| MCG-sonicate $10^4$ | 5.41 | 30.0 |
| MCG-sonicate $10^5$ | 0.00 | 100.0 |
| SOD 0.625 U | 7.16 | 7.3 |
| SOD 2.25 U | 5.12 | 33.0 |
| SOD 5 U | 2.21 | 71.4 |
| SOD 10 U | 0.69 | 91.9 |

Mast cell granules were sonicated in 50 mM sodium phosphate containing 0.5% Triton X-100 (pH = 7.0) for 2 × 20 s. The sonicates were centrifuged at 15 000 × *g* for 30 min and the supernatants were assayed for SOD activity using xanthine/xanthine oxidase system. Each value given is the mean of duplicate determinations. Commercially available SOD was used as the standard. A comparable volume of sodium phosphate containing 0.5% Triton X-100 (MCG sonication buffer) did not have any inhibitory effect on xanthine/xanthine oxidase-mediated cytochrome c reduction. Mast cell granule-sonicate is expressed as equivalent to the starting number of intact mast cells.

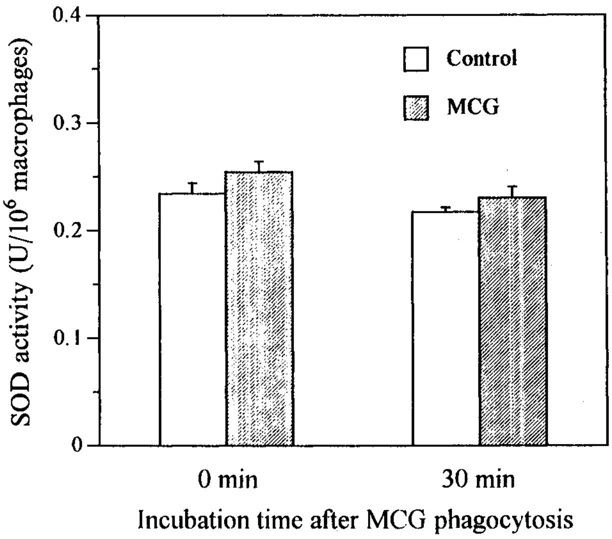

FIG. 5. SOD activity in normal macrophages and macrophages containing phagocytosed MCGs. 35 × $10^6$ macrophages were incubated with or without 3.5 × $10^6$ MCG (in mast cell equivalents) for 15 min at 37°C. The unphagocytosed MCGs were removed by gradient density centrifugation with 0.35 M sucrose. The macrophages were then washed and incubated for the indicated time at 37°C. The cells were washed and disrupted by sonication in 50 mM sodium phosphate containing 0.5% Triton X-100. The macrophage sonicates were assayed for SOD activity as described in the Materials and Methods section. Values are mean ± S.E.M., $n$=4.

rophages incubated for 30 min was completely abrogated when the MCG-sonicate was removed by washing prior to activation with PMA. The inhibitory effect was not attributable to a decrease in macrophage viability since co-incubation of macrophages with MCGs for up to 24 h did not increase the uptake of trypan blue or the release of lactate dehydrogenase activity.[15] Furthermore, MCG treatment also did not reduce the protein content of adherent cells, indicating that MCG-proteases did not affect the total number of macrophages present (data not shown).

Superoxide production by the phagocytic cells is initiated by the membrane bound respiratory burst enzyme NADPH oxidase.[7] NADPH oxidase is dormant in resting phagocytes and its activation by external stimuli involves translocation and assembly of cytosolic components to the plasma membrane.[26–28] Since MCGs are known to modulate many membrane-associated functions of macrophages such as Fcγ receptor-mediated phagocytosis,[25] LPS binding[29] and nitric oxide production,[15] it is possible that MCGs could alter the activity of NADPH oxidase as well. Nevertheless, the reversal of MCG-sonicate treated macrophages to 100% superoxide generating potential after washing indicates that MCG-sonicate did not alter NADPH oxidase activity. Since the inhibitory effect of MCG-sonicate was evident only when present in the assay system (and not just by prior treatment) the effect can be attributed to the scavenging of superoxide by SOD present in the MCGs.[24] This result also emphasizes that soluble SOD is not internalized, since washing of macrophages after incubation with MCG-sonicate or SOD completely abrogated the inhibitory effect. Further support for the presence of SOD in MCGs was that MCG-sonicate was capable of scavenging superoxide produced by xanthine and xanthine oxidase. The effect cannot be attributed to other MCG components such as histamine, serotonin or heparin since comparable concentrations of these substances did not affect the ability of macrophages to generate superoxide (data not shown).

The effect of intact MCGs on macrophages demonstrates an association between phagocytosis of MCGs and impairment of the superoxide release. When macrophages were incubated with intact MCGs for 5 or 30 min, superoxide production was decreased by 98% or 67% respectively. After the incubation, when the un-phagocytosed MCGs were removed by washing prior to activation by PMA, only partial recovery of superoxide generation (25% and 50%) was noted (Fig. 3). This could be attributed to the ability of macrophages to rapidly phagocytose SOD containing MCGs and to retain them transiently in the phagolysosomes. The partial recovery of superoxide production by MCG-treated macrophages in 30 min is consistent with the electron microscopy studies (Fig. 4) which demonstrate that macrophages rapidly phagocytosed MCGs into the phagolysosomes (Fig. 4B) and were gradually degraded or exocytosed (Fig. 4C and 4D). This may explain the partial recovery of superoxide producing capacity of MCG-treated macrophages when washed after 30 min incubation. The failure to regain 100% capacity for superoxide production in these cells may also be due to other intracellular events leading to inhibition of NADPH oxidase. It is undetermined whether degradation of SOD or breakdown of other granule components which alter NADPH oxidase assembly best explains the time- and wash-dependent recovery of macrophage superoxide production.

The present results clearly demonstrate that macrophages rapidly phagocytose MCGs into the phagolysosomes where the SOD contents of the MCGs are utilized to scavenge the generated superoxide. Subsequently, MCGs are degraded and SOD is either secreted out of the cell or is inactivated. It is noteworthy that the SOD content of macrophages treated with MCGs was not significantly higher than in control macrophages although one would expect such an increase owing to the contribution of SOD by MCGs. It is possible that although SOD from the phagocytosed MCGs may affect the generated superoxide levels in the phagosomes, their relative contribution to the total macrophage SOD may be minimal. The mechanism by which MCGs are phagocytosed is not currently known. One possible mechanism is that the uptake of MCGs is via a receptor mediated phagocytosis. It is not known if this receptor is specific or promiscuous. Whatever may be the mechanism, it is significant that MCGs are capable of regulating the phagocyte oxygen radical metabolism. The ability of MCGs to interact with and alter macrophage functions may have an important physiological role in preventing reactive oxygen-mediated tissue injury. Thus, the adverse effect of excessive mast cell degranulation could prevent phagocytes from killing invading pathogens via reactive oxygen radical dependent mechanisms.

### References

1. Adams DO, Hamilton TA. The cell biology of macrophage activation. *Ann Rev Immunol* 1984; **2:** 283–318.
2. Adams D, Hamilton TA. Molecular basis of macrophage activation. In: Lewis CE, J O'D McGee, eds. *The macrophages.* New York: Oxford University Press, 1992; 75–114.
3. Iyer G, Islam DMV, Quastel JH. Biochemical aspects of phagocytosis. *Nature* 1961; **192:** 534–541.
4. Johnston RB, Kitagawa S. Molecular basis for the enhanced respiratory burst of activated macrophages. *Fed Proc* 1985; **44:** 2927–2932.
5. Babior BM, Kipes RS, Curnette JT. Biological defense mechanism. The production by leukocytes of superoxide, a potential bactericidal agent. *J Clin Invest* 1973; **52:** 741–744.
6. Johnston RB, Keele BB, Misra HP. The role of superoxide anion generation in phagocytic bactericidal activity. *J Clin Invest* 1975; **55:** 1357–1372.
7. Babior BM, el Benna J, Faust LP. The phosphorylation of the respiratory burst oxidase component of the p47phox during neutrophil activation. *J Biol Chem* 1994; **38:** 23431–23436.
8. Halliwell B, Gutterridge JMC. Oxygen free radicals and iron in relation to biology and medicine: some problems and concepts. *Arch Biochem Biophys* 1986; **246:** 501–514.
9. Dileepan KN, Stechschulte DJ. Influence of mast cells on macrophage function. *Biochem Soc Trans* 1986; **14:** 913–914.
10. Dileepan KN, Simpson KM, Stechschulte DJ. Modulation of macrophage superoxide-induces cytochrome c reduction by mast cells. *J Lab Clin Med* 1989; **5:** 577–585.
11. Dileepan KN, Simpson KM, Lynch SR, Stechschulte DJ. Dismutation of eosinophil superoxide by mast cell granule superoxide dismutase. *Biochem Arch* 1989; **5:** 153–160.
12. Yurt RW, Leid RW, Spragg J, Austen KF. Immunologic release of heparin from purified rat peritoneal mast cells. *J Immunol* 1977; **4:** 1201–1207.
13. Stechschulte DJ, Austen KF. Phosphatidylserine enhancement of antigen-induced mediator release from rat mast cells. *J Immunol* 1974; **12:** 970–978.
14. Raphael GD, Henderson WR, Kaliner M. Isolation of membrane-bound rat mast cell granules. *Exp Cell Res* 1978; **115:** 428–431.
15. Dileepan KN, Lorsbach RB, Stechschulte DJ. Mast cell granules inhibit macrophage mediated lysis of mastocytoma cells (p815) and nitric oxide production. *J Leukoc Biol* 1993; **53:** 446–453.
16. Dileepan KN, Kennedy J. Complete inhibition of dihydro-orotate oxidation and superoxide production by 1,1,1-trifluoro 3 thenocylacetone in rat liver mitochondria. *Biochem J* 1985; **225:** 189–194.
17. McCord JM, Fridovich I. The utility of superoxide dismutase in studying free radical reaction. I. Radicals generated by the interaction of sulfite, dimethyl sulfoxide and oxygen. *J Biol Chem* 1969; **244:** 6049–6055.
18. Janes J, McDonald JR. Mast cells. Their distribution in various human tissues. *Arch Path* 1948; **45:** 622–632.
19. Crisp AJ, Chapman CM, Kirkham SE, Schiller AL, Krane SM. Articular mastocytosis in rheumatoid arthritis. *Arthritis Rheum* 1984; **27:** 845–851.
20. Godfrey HP, Ilardi C, Engber W, Granciano FM. Quantitation of human synovial mast cells in rheumatoid arthritis and other rheumatic diseases. *Arthritis Rheum* 1984; **27:** 852–856.
21. Bromley M, Fisher WD, Woolley DE. Mast cells at sites of cartilage erosion in the rheumatoid joint. *Ann Rheum Dis* 1984; **43:** 76–79.
22. Yoffe JR, Taylor DJ, Woolley DE. Mast-cell products and heparin stimulate the production of mononuclear-cell factor by cultured human monocyte/macrophages. *Biochem J* 1985; **230:** 83–88.
23. Lindahl U, Pertoft H, Seljelid R. Uptake and degradation of mast-cell granules by cultured fibroblasts. *J Immunol* 1983; **182:** 189–193.
24. Subbarao PV, Friedman MM, Atkins FM, Metcalfe DD. Phagocytosis of mast cell granules by cultured fibroblasts. *J Immunol* 1983; **130:** 341–349.
25. Yamada A, Dileepan KN, Stechschulte DJ, Suzuki T. Regulation of Fc2γ a receptor-mediated phagocytosis by a murine macrophage-like cell line p388D1. Involvement of casein kinase 11 activity associated with Fcγ2 a receptor. *J Mol Cell Immunol* 1989; **4:** 191–202.
26. Clark RA, Volpp BD, Leidal KG, Nauseef WM. Two cytosolic components of the human neutrophil burst oxidase translocate to the plasma membrane during cell activation. *J Clin Invest* 1990; **85:** 714–721.
27. Segal AW, Abo A. The biochemical basis of the NADPH oxidase of phagocytes. *TIBS* 1993; **18:** 43–47.
28. Chanock SJ, el Benna J, Smith RM, Babior BM. The respiratory burst oxidase. *J Biol Chem* 1994; **40:** 24519–24522.
29. Dileepan KN, Lei MG, Morrison DC, Stechschulte DJ. Inhibition of macrophage-mediated tumor cell killing by mast cell granules. Evidence for modulation of LPS binding to receptor. *J Leukoc Biol* 1990; **1** (Suppl): 48.

ACKNOWLEDGEMENTS. This work was supported by grants from the American Heart Association (Kansas Affiliate, #KS-92-GS-36), American Federation of Aging Research, and Joseph G. and Elizabeth E. Carey Arthritis Research Funds. We are grateful to Jordan Page for his technical assistance. We acknowledge the services from The Electronmicroscopy Research Laboratory at Kansas University Medical Center.

**Received 4 July 1995; accepted 15 August 1995**
